# Cultural adaptation of a shared decision making tool with Aboriginal women: a qualitative study

**DOI:** 10.1186/s12911-015-0129-7

**Published:** 2015-01-28

**Authors:** Janet Jull, Audrey Giles, Minwaashin Lodge, The Aboriginal Women’s Support Centre, Yvonne Boyer, Dawn Stacey

**Affiliations:** Bruyère Research Institute & University of Ottawa, 85 Primrose Avenue, Room 312, University of Ottawa, Ontario K1R 7G5 Canada; Faculty of Health Sciences, School of Human Kinetics, University of Ottawa, 334 Monpetit, Ottawa, Ontario K1N 6 N5 Canada; Box 181, Merrickville, Ontario K0G 1 N0 Canada; Faculty of Health Sciences, School of Nursing, University of Ottawa, 451 Smyth Road, Ottawa, Ontario K1H 8 M5 Canada

**Keywords:** Equity, Aboriginal, Indigenous, Women, Shared decision making, Cultural adaptation, Usability testing, Health literacy

## Abstract

**Background:**

Shared decision making (SDM) may narrow health equity gaps experienced by Aboriginal women. SDM tools such as patient decision aids can facilitate SDM between the client and health care providers; SDM tools for use in Western health care settings have not yet been developed for and with Aboriginal populations. This study describes the adaptation and usability testing of a SDM tool, the Ottawa Personal Decision Guide (OPDG), to support decision making by Aboriginal women.

**Methods:**

An interpretive descriptive qualitative study was structured by the Ottawa Decision Support Framework and used a postcolonial theoretical lens. An advisory group was established with representation from the Aboriginal community and used a mutually agreed-upon ethical framework. Eligible participants were Aboriginal women at Minwaashin Lodge. First, the OPDG was discussed in focus groups using a semi-structured interview guide. Then, individual usability interviews were conducted using a semi-structured interview guide with decision coaching. Iterative adaptations to the OPDG were made during focus groups and usability interviews until saturation was reached. Transcripts were coded using thematic analysis and themes confirmed in collaboration with an advisory group.

**Results:**

Aboriginal women 20 to 60 years of age and self-identifying as First Nations, Métis, or Inuit participated in two focus groups (n = 13) or usability interviews (n = 6). Seven themes were developed that either reflected or affirmed OPDG adaptions: 1) “This paper makes it hard for me to show that I am capable of making decisions”; 2) “I am responsible for my decisions”; 3) “My past and current experiences affect the way I make decisions”; 4) “People need to talk with people”; 5) “I need to fully participate in making my decisions”; 6) “I need to explore my decision in a meaningful way”; 7) “I need respect for my traditional learning and communication style”.

**Conclusions:**

Adaptations resulted in a culturally adapted version of the OPDG that better met the needs of Aboriginal women participants and was more accessible with respect to health literacy assumptions. Decision coaching was identified as required to enhance engagement in the decision making process and using the adapted OPDG as a talking guide.

## Background

Delivery of care from within traditional Western healthcare models often undermines Aboriginal peoples’ health and well-being as these care models reflect values, the use of knowledge systems, and care practices that may not align with those of Aboriginal people [1,2]. Western-trained health care providers typically lack understandings of diverse Aboriginal cultures [1,3], which has had a negative impact on the health of Aboriginal women [4] and affected their participation in health care settings [5].

Aboriginal women have a right to safe and effective care practices, including participation with health care providers in making meaningful decisions about their health. Shared decision making (SDM) is a process of collaboration between health care providers and clients, developed within Western-informed health care settings [6]. SDM has been found to increase the client’s level of satisfaction with care decisions by better meeting client’s information needs and incorporation of client’s values into health care decisions [7,8]. In summary, SDM is central to patient-centred care [9]. Evidence derived from studies conducted with Aboriginal people about SDM in health care settings is limited [10]. Our previous study indicated that Aboriginal women view SDM as including relational features and which are identified as core competencies for SDM [11] although these views are not yet evident in mainstream models of SDM or in SDM tools and approaches [12].

SDM is facilitated by patient decision aids and decision coaching to support decision making that is shared between health care provider(s) and client [13]. Patient decision aids can facilitate the sharing of information and can contribute to helping the client make preference sensitive decisions by informing the client of the benefits and harms of care options [14]. Patient decision aids are booklets, videos, or online tools that complement practitioner counseling; they have been found to increase people’s involvement in making more informed and value-based care decisions. Although there are over 300 publicly available decision aids, there is much overlap on topics and there remain many decisions for which patient decision aids have not yet been developed. In addition, none of these decision aids have been deemed culturally appropriate or defined as adequate for all Aboriginal populations.

Decision coaching supports SDM and coaches are trained to be non-directive, to provide evidence, and to support people rather than offer advice, so that people make choices consistent with their own values and beliefs [15,16]. In addition, decision coaching tailors decision support to be relevant to each situation and is aimed at building decision making skills so that people can apply these skills in other situations. Used alone, decision coaching has been found to improve knowledge for clients and, when combined with a patient decision aid, to increase knowledge and participation in care [16].

Participation in health care requires health literacy skills, which are described as the ability to access and use care, the ability to understand and use information for health and well-being, and the capacity to use information effectively. High levels of health literacy result in empowerment and the capacity to make decisions that support favourable health outcomes for the individual participating in health care systems [17]. Health literacy issues have been identified as barriers to participation in decision making and to attaining the best outcomes with health services [18,19]. There have been a number of issues described, which undermines the health literacy of a range of populations, including Aboriginal women [20]. For instance, due to historical factors that have created societal inequities and/or limited access to educational resources, Aboriginal learners have lower graduation rates and are less likely to be in age appropriate grades [21]. As well, Aboriginal populations in Canada must deal with the complexity of cultural identity legislation and other challenges that undermine their ability to negotiate systems of health care; this often leads to limited access to or exclusion from health and social programs [22]. These are some of the factors that challenge the health literacy of Aboriginal people and may ultimately disrupt their ability to be equitable participants in decision making.

Currently, there are no studies of SDM tools that have been developed for and with Aboriginal populations for use within Western health care settings. Given the social systems and structures that undermine health and well being of this population, it is imperative that research be conducted in collaboration with Aboriginal people as equal partners to explore and adapt current approaches to SDM that are culturally relevant for Aboriginal populations who are accessing mainstream health care services. Prior to conducting this research project, we engaged in a series of studies with an advisory group and Minwaashin Lodge [10,12,23]. These studies affirmed the decision to engage in a process of adaptation and usability testing of a patient decision aid. While guidelines that outline the cross-cultural adaptation of self-report measures exist [24], for our study we chose to support the adaptation of a patient decision aid from within a mutually agreed upon partnership and ethical framework, and using a process aligned with the socio-cultural values of those in the partnership. This study is the result of a partnership with an Aboriginal women’s organization, Minwaashin Lodge, and was conducted in complete collaboration with members of the study’s advisory group, all of whom were decision makers in the study and are co-authors of this paper. The ideas in this paper are the result of work developed from the interests of the first author (JJ), a Euro-Canadian woman who has had years of experience working with and learning from Aboriginal people in clinical settings of urban, rural, and remote regions of Canada. During her doctoral research studies, she developed ideas leading to this study in efforts to identify and address systems-level issues that undermine the health of Aboriginal people, through working closely with an advisory group; experts in the area of shared decision making and knowledge translation (DS); qualitative methods and research with Aboriginal people (AG); the law, research, and Aboriginal people (YB); the status of Aboriginal women and children who are at risk of or who have experienced violence (leaders at ML). The study research partnership with Minwaashin Lodge, a community-based organization that provides services (e.g., shelter, counseling, training programs) to First Nations, Métis, and Inuit women and children who are survivors of family violence and/or the residential school system, was an integral and sustaining feature of this work. Minwaashin Lodge leaders viewed this study as of potential benefit to its community of women and children, both as an opportunity to talk about experiences of importance to them and as an opportunity to potentially influence health care systems. The purpose of this study is to describe the adaptation and usability testing of the Ottawa Personal Decision Guide (OPDG) to support decision making by Aboriginal women.

### Theory

Two distinct theoretical perspectives were selected to support and/or align with the ethical framework and used to inform this study: the Ottawa Decision Support Framework [25] and postcolonial theory [21].

The Ottawa Decision Support Framework (ODSF) informs the study. The ODSF is an evidence-based, practical, midrange theoretical framework developed to guide people through health and social decisions and incorporates three key elements: decisional needs, decision support, and decision quality [25]. According to the framework, unresolved decisional needs will negatively influence decision quality. While it has not been used specifically with Aboriginal populations, the ODSF has been successfully used to structure the assessment of decisional needs within a range of populations in Canada and internationally [25] and specifically with women [26,27]. The ODSF provided the theoretical foundation for the patient decision aid adapted in this study and was used with postcolonial theory to guide the creation of key questions and prompts for focus groups and usability interviews. The ODSF provides a theoretical framework for structuring SDM tools and approaches (i.e., patient decision aids, decision coaching) and was used with a postcolonial theoretical lens through which SDM was viewed for this study.

Postcolonial theory encompasses a group of theories that share a social, political and moral concern about the history and legacy of colonialism and are derived from diverse disciplinary perspectives [28,29]. An essential feature of postcolonial theory, and of particular relevance to the work described here, is a focus on disrupting the thinking behind structural inequities, such as those that are evident in health care systems, that have been brought about by the histories and ongoing legacy of colonial practices [3]. Aboriginal scholars have made strong contributions to postcolonial thought; these contributions have developed from Aboriginal epistemologies and the need to accommodate the complexities of identifying and seeking to address colonialism [22]. A postcolonial perspective provides a theoretical lens to show how marginalization occurs in day-to-day relationships and in the systems structuring human relations, such as the health care setting [30,31].

The approach for this study is underpinned by Battiste’s [21] articulation of postcolonial theory. She describes the need for transformative strategies from which to understand and strive to resolve the range of issues experienced by Aboriginal people and their communities related to oppression and marginalization that results from colonization. Battiste [21] situated Aboriginal people as central to a collaborative process of societal change with non-Aboriginal people. This perspective aligns with this study as it was developed from a research partnership between the study’s first author and Minwaashin Lodge, an Aboriginal led organization that serves Aboriginal women. Battiste’s [21] postcolonial theory principles were adopted for this study as the most appropriate lens through which to view and address the complex intersections of colonialism’s impact on the lives of the Aboriginal women [30,31] who participated in the study. The use of Battiste’s postcolonial lens [21] ensured that those conducting the study worked towards implementing research processes that examined approaches to SDM while promoting a decolonizing agenda. For example, there was ongoing reflection on study practices and adherence to the ethical framework by the first author and the advisory group throughout the study. The postcolonial lens also guided the data analysis phase to evoke the complex and interacting political, social, and historical factors that influence women’s use of a shared decision making tool like the OPDG, which led to adaptations that are described later in this paper.

## Methods

### Design

An interpretive descriptive qualitative study design was selected for this project as previous researchers have identified it as an effective method for describing health events [32]. For this study, the interpretive descriptive approach supported an iterative process occurring between data collection and data analysis; the use of informed questioning of participants by the researcher; participant and researcher reflection and examination of ideas; and the creation of an interpretive account of what was studied [32]. The interpretive descriptive process directed the study and the adaptations made to the OPDG. This qualitative approach also supported the generation of new ideas during the study [33] and aligned with postcolonial theory and the ethical framework used to structure this study. Interpretive description is a practical and accessible approach that we used to build knowledge by linking information from Aboriginal women at Minwaashin Lodge about health decision making experiences with information derived from broader knowledge systems, such as those historical, political and social structures, which influence health systems access by Aboriginal people. Further, it supported the development of new understandings about Aboriginal women’s preferences for an SDM tool. This study was approved by the University of Ottawa’s Research Ethics Board, and also received ethical approval from Minwaashin Lodge Executive and leaders. An ethical framework was developed by the study advisory group, whose membership included those of Aboriginal and of Euro-Canadian descent, and was structured by guidelines for ethical research with Aboriginal people [33,34]. The ethical framework was designed to support a research agenda respectful of the diverse needs of a population of Inuit, First Nations and Métis women, and also reflected in a memorandum of understanding. The study protocol provides details on the study partnership and the ways in which Aboriginal understandings of health and well-being were incorporated into the original design of the study, and was published a priori [23].

### Setting and participants

Minwaashin Lodge representatives directed potential participants to recruitment posters and/or provided contact information to solicit information from the first author (JJ), and in this way participants were purposefully recruited for the study. Women who participated in the focus groups were not eligible for participation in the usability interviews. Participant inclusion criteria were those who self-identified as Aboriginal women, that were 18 years or older, were clients of Minwaashin Lodge, and were able to participate in an interview conducted in English.

### Intervention to be adapted: the OPDG

The OPDG [35] is a generic tool that was developed according to the ODSF and is used by people to help self-assess decisional needs, summarize knowledge, clarify values, and plan next steps when making any social or health decision. It can also be used as an adjunct to coaching by a care provider [24], and in response to focus group feedback was used with decision coaching during usability testing interviews. Although not yet evaluated for use by Aboriginal populations, the OPDG has been validated for use with general populations [25], and, more specifically, with Japanese and American women considering treatment options [36,37].

### Procedure

**Figure 1:**
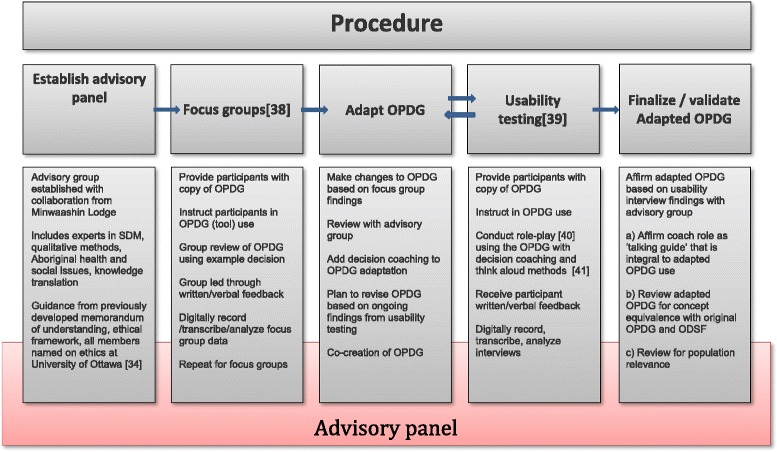
**Procedure.**

The procedure for OPDG adaption and usability testing is presented in Figure 1 [38-41]. Written informed consent was sought and obtained from all participants. The first author (JJ) and a research assistant (CD) facilitated 2 focus groups in which participants indicated whether the patient decision aid was acceptable or not, and if not, what changes they would recommend. Then, the usability testing was conducted with decision coaching by the first author (JJ), who is a trained decision coach. The semi-structured interview guides were developed using the ODSF and postcolonial theory and in collaboration with Minwaashin Lodge. An example decision about a return to school was selected as a neutral, non-distressing social decision, and was identified as a common experience by Minwaashin Lodge representatives (Tables 1 and 2). At the completion of the usability interviews, the final version of the adapted OPDG was reviewed by the first author (JJ) and with an OPDG developer (DS) to ensure concept equivalence between the original and adapted OPDG, and then with representatives of Minwaashin Lodge for population relevance.

**Table 1:** **Examples of questions asked by interviewer of focus group participants**

**Question**	**Prompt**
1. In general, what did you like or not like about this form? Was anything confusing about it?	Made sense? Seems organized? Useful? Why/why not?
2. Do you think that this form would be useful when considering a decision about your health and well being, or about something like whether to go to school?	Do they seem to ask the right questions? Do the topics/ideas seem right?
3. We will now go through each question on the form. Do you think that #_ makes sense?	If a concern is raised: What do you like/not like? What would you change?
4. Do you have any other comments or suggestions that we should consider that would make the form easier for Aboriginal women to use?	Topics, ideas: particular words, pictures?
5. Those are all the questions we were going to ask; would you like to ask us about anything? Is there anyone else you think we should talk with about this topic?	
6. Do you think we have created a tool that could be useful for making decisions about health?	
7. Would you try it out again for making a real decision?	Why or why not? What was it like to use? What would make it better?
8. Do you think that this could help you to make a decision that you think is good?	Clarify the choice options? Figure out the benefits and harms? The chances that the benefits or harms might happen?

**Table 2:** **Examples of questions asked by interviewer of usability participants**

Background Statement (interviewer speaking to participant): ‘This is your decision scenario – so I am asking you to pretend to be preparing to go in to see your care provider, a counsellor, social worker, doctor/nurse, to make a decision about a return to school’.	
The participant talks through how she would use the OPDG to prepare for her meeting with a care provider, and answers some brief questions at the end of the role-play on her views towards using the OPDG.	
**Question**	**Prompts**
1. Was the OPDG easy to use?	Did it make sense the way it was organized? Was it clear?
2. Would you try it out again/for making a real decision?	Why or why not? What was it like to use it? What could make it better?
3. Do you think that this could help you to make a decision that you think is good?	Clarify the choice options? Figure out the benefits and harms? The chances that the benefits or harms might happen?
4. Do you have any ideas on what might help you to be more involved in decisions and choose what you think are better options?	

### Data analysis

Transcripts of focus group and usability interviews underwent thematic analysis. A six phase process was used for thematic analysis [42]: 1) familiarization with data; 2) generation of initial codes within each transcript (e.g. “hard to read/understand”; “responsible for decisions”; 3) search for themes (e.g. “paper is hard to understand”; “I take my decisions seriously”; 4) review of themes; 5) define and name themes, which were further confirmed or adjusted by a second reviewer (CD); 6) reporting of themes in a way that reflected the rationale for the adaptations to the OPDG. At each phase, the advisory group was engaged for feedback, with one person (JJ) central to the process and other members (AG, ML, YB, DS) having the process described to them and/or contributing throughout the process (Figure 1). This process supported the principles outlined in the RATS guideline, and which were adhered to for quality reporting of the study [43].

Throughout the thematic analysis, findings were examined using a postcolonial theoretical lens by situating them in a social, historical, and political perspective [21]. At the completion of the usability-testing interviews, and following final confirmation by interview participants of the adapted OPDG acceptability, the adapted OPDG and final findings were reviewed and confirmed in collaboration with Minwaashin Lodge leaders and with the rest of the advisory group.

## Results

### Participant characteristics

Nineteen Aboriginal women participated in the study in 2 focus groups (n = 13) or usability interviews (n = 6) (Table 3). Participants self-identified as First Nations, Métis, or Inuit women, between the ages of 20 and 60 years and with education ranging from grade 8 to university and/or college levels. Many of the participants were responsible for the care of children, Elders, or extended family. The names and identifying characteristics of the study participants have been changed to preserve anonymity.

**Table 3:** **Demographic data for focus groups and usability interviews**

**Demographic data**	**Participants (N = 19)**
Inuk	2
First Nations	7
Métis	10
Age Range	
20 to 29	7
30 to 49	9
50 to 50	3
Number of children:	
0	2
1	2
2	6
3	4
4	3
5	2
Education:	
<Grade 8	2
Grade 8 to 12	13
College	1
University	3

### OPDG adaptation

**Figure 2:**
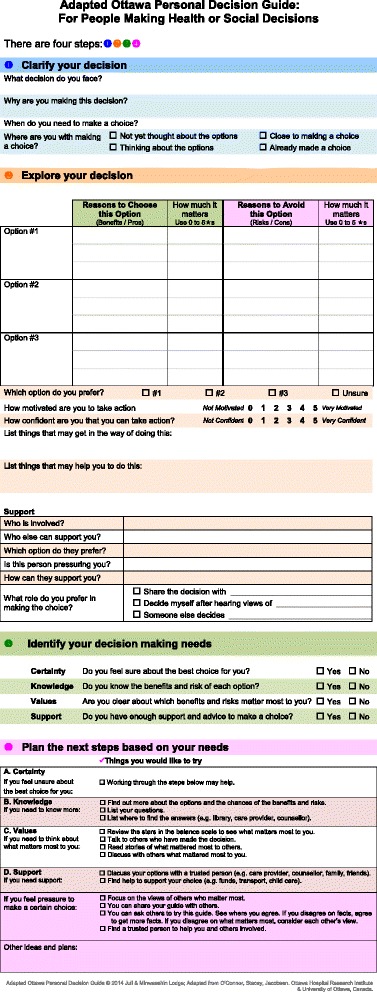
**Adapted Ottawa personal decision guide.**

Focus groups and usability interview participants suggested OPDG adaptations (Themes 1 through 4, Table 4) and confirmed the relevance of the adapted OPDG when used with decision coaching (Themes 5 through 7). The adapted OPDG is presented in Figure 2.

**Table 4:** **Themes informing OPDG adaptations**

**Theme**	**Adaptation to OPDG**	**Focus groups**	**Usability testing**
“This paper makes it hard for me to show that I am capable of making decisions”	• Plain language	?	?
	• Print size	?	
	• Decreased concept density	?	?
	• Logical layout	?	?
“I am responsible for my decisions.”	• Addition of text in ‘Support’ (section 2)	?	
	• Removal of extra text (section 3)	?	?
	• Positive language (section 4)	?	?
“My past and current experiences affect the way I make decisions.”	• Use of neutral language and/or meaningful language	?	?
	• Addition of 4 lines for decision implementation (section 2)	?	
	• Tailored to population – less like ‘government form’	?	?
“People need to talk with people”	• Coach facilitates access of OPDG, meaningful use of OPDG, integrates context into use of OPDG process	?	?

### Theme 1: “This paper makes it hard for me to show that I am capable of making decisions”

The theme reflects the OPDG adaptations to support participant ability to obtain, understand, and use factual information. The theme reflects the needs of participants to have accessible, user-friendly tools. As several participants reported, their experience with Western care systems and settings did not foster their full participation when accessing and using care. During the initial iterations of the OPDG adaptation, participants were observed to often be looking silently at the paper until asked their views on the readability of the OPDG. Myrna described her difficulties with the OPDG when she stated, “It’s a little confusing, okay. The lists – it does not make sense.” Others voiced similar issues, with comments such as “I am not sure of what this means” or sometimes simply asking the facilitator, “what should I put?” To address these issues, participants identified several essential adaptations: a) use of plain language; b) adjustments to print size to better identify transitions to each new section; c) decreases to concept density (creation of extra white space by decreases in text density throughout text; provision of space for notes in section 4; removal of repetitive wording in section 4); d) a more logical layout of the OPDG text (alignment of the section 3 list with section 4, boxing lists in section 4 together to make information appear more manageable).

### Theme 2: “I am responsible for my decisions”

The theme reflects adaptations aimed at enhancing participants’ understanding of facts, enabling meaningful communication with health care providers, and helping participants to use information to meet their particular needs. Participants were found to be sensitive to the wording in the OPDG; they explained that some of the wording undermined their autonomy in care settings. In Section 2, participants identified the concept of ‘support’ as problematic and as implying that others should be making the decision for them. As Leah stated, “the decision is mine alone to make.” Some participants, such as Dana, related challenges to ensuring that she was not obstructed in her decision making processes: “I have some people that I know that are doing that [are trying to tell me what to do] - and I say you cannot make my choice. I am the one who has to make my own choice.” Miriam provided some additional insight into the concept of support: “Support – who is involved? There is no one but me. I would like someone…but I don’t have anyone; everyone’s gone.” Participants also recognized that others could or should sometimes be involved in their decision making processes, and that in every situation their decision making affected others. For this reason, an extra line of text was added to broaden the concept of support and to make it potentially more culturally relevant to participants (Section 2: ‘Who else can support you?’).

Participants also indicated that the language of the OPDG was sometimes negative and was not aligned with the approach or attitude towards decision making. Further, some participants stated that the language seemed to reflect the discrimination they often perceived in care settings. For instance, the statement that defined the results of test questions that screen for decisional conflict in Section 3 (the ‘SURE test’) was perceived as blaming the person using the form for not having enough certainty, knowledge, values, or support when decision making. Therefore, this statement was removed (‘People who answer “No” to one or more of these questions are more likely to delay their decision, change their mind, feel regret about their choice or to blame others for bad outcomes.’). The changes reflected preferences expressed by participants to avoid feeling that they were being directed in their decision making. Instead, participants indicated that their preference was to participate in a self-directed process of decision making that supported a more familiar approach to problem solving. For instance Sarah said, “When I want something…need to get information, I have options, there is always a way…I can figure it out. I don’t need someone else telling me what I can’t do, don’t know”.

In addition, phrases were reframed using positive language, evident in a list of choices in Section 4 (for example, participants suggested ‘If you need to know more’ in place of ‘If you feel you do NOT have enough facts’ et cetera). Section 4 of the OPDG was further reworded during interviews to reflect the role of trusting oneself and others during decision making, for participants expressed concern about assumptions within care relationships, described by Anna: “I just balked at being told to ‘share your guide with others’ and ‘ask others to complete this guide’. And - I have heard that you can go to a neutral person, but how can you know them well enough? You have to build trust.” The language was also made more reflective of Aboriginal women’s approach to decision making. For instance, changes were made to make the language less directive (for example, ‘Ask others’ became ‘You can ask others’) and more personable (for example, ‘Find a neutral person’ became ‘Find a trusted person’).

### Theme 3: “My past and current experiences affect the way I make decisions”

The third theme represents adaptations to the OPDG to support reflection by participants on information or advice received, including the influence of wider social determinants of health. The factors that influence people’s access and use of care services cannot be separated from the socio-historical contexts in which they are situated; participants identified this during the OPDG adaption. Changes to the OPDG were several-fold: using language defined by participants as meaningful, adding four decision implementation questions to Section 2, and tailoring the look of the OPDG to appeal to the participant population. Participants talked about how the use of language was important not just for readability, but for feelings of engagement with the decision making process for, as Eliza-Jane said, “The words that are used here – it sounds just like another survey. We don’t need any more surveys – we need resources that we can actually use to actually help.” Changes to language throughout the text were made to not only reflect use of plain language, but also language familiar to women (for example, removing language perceived by participants as more technical and directive throughout the OPDG, and substituting more personable and familiar terms such as that of ‘care provider’ instead of ‘health provider’ in section 4).

The addition of four lines to the OPDG to include questions identifying implementation needs (Section 2) was affirmed by participants who talked about resource and personal barriers to carrying out decisions, (the extra four lines included the following: ‘How motivated are you to take action’; ‘how confident are you that you can take action’; ‘list things that may get in the way of doing this’; list things that may help you to do this’). Maeve stated, “making the decision is one thing; doing it is another” when talking about how health care providers rarely seemed to want to talk about or understand the situational barriers experienced by Aboriginal women making care decisions (e.g., lack of childcare, funding, transportation). Participants viewed the ‘doing’ of the decision as an integral part of the ‘making’ of the decision and described the process of decision making as situated within social, historical, political systems which often acted as barriers to implementation of their decisions.

The OPDG was also critiqued as looking like a ‘government form’. One participant noted that Aboriginal women “have had forms used against them” within social, historical and political systems. Participants suggested showing Aboriginal affiliation on the form (for example, Minwaashin Lodge’s logo and name), as well as further colour and spacing changes, and with potential for further tailoring (e.g., additional graphics, affiliations) in order to make the form more appealing to other clients of Minwaashin Lodge.

### Theme 4: “People need to talk with people”

Participants identified that they wanted a person knowledgeable with the OPDG to play a role in their use of the OPDG. This theme describes the impact that supportive interactions can have on people that experience marginalization within care and social systems. As described by Melissa, “this would not work as it is, as a paper you give to someone. To make a decision, it’s personal…for example, my aunty would not use this – older people, others who do not use forms much – they like to talk. That is how they make their decisions.” Participants described their views that the OPDG should be a supporting element of a broader strategy, involving a trained person (decision coach) who could assist women in obtaining and understanding information and to provide support and build confidence with women such that they could use the information in a way they defined as meaningful and which accurately reflected their context. Sixteen of the 19 participants said that they would consider using the adapted OPDG in the future, but significantly, only 1 of the sixteen stated that they would consider using it *without* a coach. For those (n = 3) who said they would not use the adapted OPDG, their reasons were that they felt it was too much like a government form (one participant), and that decisions are too personal to make using a form (two participants).

### Theme 5: “I need to fully participate in making my decisions”

This theme reflected the participants’ engagement in the decision making process and reflected their growing confidence as they became more proficient in the use of the adapted OPDG with the support of their decision coach. During interviews, participants related experiences in which they expressed frustration and anxiety leading to low confidence about being able to receive help needed from health care providers, and their low expectations about positive care experiences. For instance, following the coach’s introduction of the adapted OPDG, one participant, Alicia, was silent, and when asked by the coach if it was okay to start, Alicia stated, “I am a good reader” and continued to remain silent. This was interpreted by the coach to mean that the participant required additional support. The coach assisted Alicia with the adapted OPDG, and Alicia responded and became progressively more confident in directing the coach in the use of the adapted OPDG, and to make comments to help with further adaptation. She explained why she wanted this support: “It’s just hard to answer – I’ve never done anything like this before – a paper or making a decision like this”.

Participants affirmed the readability of the adapted OPDG, easily engaged with the text, and did not suggest further changes to influence readability of the adapted OPDG. They also identified the coaching role as an integral part of using the adapted OPDG. One participant, Samantha, explained: “[there is] the need to see that you [coach] are on my side, ready to work with me – it is a consensus process. Some people are more visual learners, and these words - they are not going to work for them. You have to be ready to make this work for everyone”.

### Theme 6: “I need to explore my decision in a meaningful way”

Participants identified the need for the adapted OPDG and decision coach to facilitate the meaningful acquisition and use of information. While participants viewed themselves as making care decisions, they indicated that in typical care settings their role(s) and way(s) of making decisions went unacknowledged and were undermined by dominant systems and social norms. The coach played a strong role in tailoring the way in which the adapted OPDG was used to foster respectful decision making processes. For instance, participants questioned the system for rating option preferences that involved scoring the values of options in relation to each other (Section 2). Glenda described the dilemma: “I cannot put stars to differentiate – they all mean a lot to me. I would want to talk about it instead.” Samantha also emphasized the importance of a conversation with a decision coach rather than making relative rankings of options: “Without this [coach-participant] conversation…this paper is just ‘do you want to do this or that’ – not the ‘why’”. These responses showed that it was undesirable for users to quantify the meanings attached to different options along with their pros and cons. Participants preferred to focus on talking through the meanings of options with the coach. When used in this new format, the participants affirmed that the adapted OPDG fostered respect for their preferred approach to decision making.

### Theme 7: “I need respect for my traditional learning and communication style”

The final theme reflects the awareness of participants for the ways in which determinants of health, such as income, education, culture, influence their participation in decision making. During the interviews, all of the participants described how complex and interacting historical, political, and social issues influenced their care experiences. For instance, Chloe described language barriers and the complexity of past historical factors (residential schooling) as influencing and creating barriers to participation for community members when working with health care providers: “In my [community], how we use language is different…and if we use it, we are considered unintelligent [gives example of how language translates into a different structure in English]. Others who are not from our community make fun of this and then…they [community members] are demeaned.” Elizabeth explained how she had experienced barriers to care, as the health care system seemed to discount or discredit Aboriginal peoples’ traditions, knowledge or perspectives: “The person with the most knowledge within an Aboriginal community may have no education, but they are much smarter than you or I”.

Participants encouraged the decision coach in a process of learning *with* them as well as *about* them and their decision making needs, for, as Samantha stated, “If you are asking for ways to make decisions with an Indigenous person, then you have to acknowledge that these social problems are there.” Participants viewed the decision coach as a person prepared to accompany them in a journey of decision making, and the decision coach was seen as a critical facilitator of decisions, one that was inseparable from the adapted OPDG form.

## Discussion

The adapted OPDG is a patient decision aid designed by and with Aboriginal people aimed at restructuring approaches to care with Aboriginal clients in Western health settings; in this study adaptations were conducted by a diverse (First Nations, Métis and Inuit) group of Aboriginal women from various parts of Canada and who are clients of Minwaashin Lodge. Focus groups and usability interviews with Aboriginal women were used to adapt, refine, and affirm the OPDG for use by decision coaches. Seven themes were identified that reflected the adaptations made by the participants to the original OPDG (Themes 1 through 4) or affirmed the adapted OPDG (Themes 5 through 7). Our findings demonstrated that adaptations resulted in a more culturally sensitive and accessible version of the original OPDG that were identified by Aboriginal women participants as better able to meet their decision making needs within Western health care settings. Additionally, decision coaching was identified by participants as a way to enhance their engagement in the decision making process using the adapted OPDG. Further, our study suggests that current health literacy frameworks may require expansion to accommodate more inclusive understandings of health literacy within various Aboriginal populations. A postcolonial theoretical lens was used to show how the adapted OPDG with coaching can support Aboriginal women as they negotiate societal disadvantages that are influenced by the political, social, and historical systems in which they must function.

### Adaptations that better meet participant needs

Our findings indicated that the adapted OPDG with coaching resulted in a version of the original OPDG that was identified by participants as better able to meet their decision making needs, a more accessible version of the OPDG that made fewer assumptions about complex English reading and comprehension skills, and built on participant strengths in the areas of interactive and critical literacy skills. The first four themes identified adaptations to the OPDG and align with Nutbeam’s [44] three tier model of Health Literacy: Theme 1 (“This paper makes it hard for me to show that I am capable of making decisions”) identified the need to enhance functional literacy skills through increasing the readability of the OPDG; theme 2 (“I am responsible for my decisions”) identified the need to facilitate the meaningful use of the OPDG, which relates to interactive health literacy skills; theme 3 (“My past and current experiences affect the way I make decisions”) identified opportunities for critical reflection and incorporation of contextual features into the OPDG; and theme 4 (“People need to talk with people”) supported health literacy at all three levels by engaging the decision coach in a supportive role with Aboriginal women using their functional, interactive and critical health literacy skills. As Nutbeam’s [44] model described health literacy as the result of complex sociocultural factors, it is appropriate for use within Aboriginal contexts; understanding and building literacy for and with Aboriginal populations has been identified as requiring the accommodation and integration of sociocultural factors, including Aboriginal views and beliefs [45]. Addressing lower levels of health literacy has been defined as crucial for decreasing disparities in health status experienced by populations [19]; our findings show one potential user group’s approach to fostering and supporting health literacy skill.

The remaining three themes were also found to align with health literacy as defined by Nutbeam [44] and reflected the role played by the decision coach to support health literacy: Theme 5 (“I need to fully participate in making my decisions”) reflected the participants’ functional health literacy skill, reflected by their ability to engage in the decision making process and use the adapted OPDG with the decision coach; theme 6 (“I need to explore my decision in a way that is meaningful to me”) identified participants’ interactive health literacy skill by using the adapted OPDG with coaching to foster decision making processes they defined as meaningful; and theme 7 (“I need respect for my traditional learning and communication style”) relates to participants’ critical literacy skills and their awareness of contextual features factoring into their decision making process when using the adapted OPDG with decision coaching. In our study the addition of decision coaching addressed health literacy skills (functional, interactive, critical), leading to a better understanding and use of information.

The adapted OPDG, of which decision coaching is a critical part, suggests an SDM strategy for Aboriginal women that may be used to inform and structure interactions with Western trained health providers. Further, it aligns with Battiste’s [21] postcolonial approach in which social interactions that underlie oppression of Aboriginal people and contribute to undermining health literacy are resolved from within a partnership. Additionally, this strategy may have broader potential applicability to other populations who face similar challenges with health literacy and inequitable barriers to access and negotiate systems of care.

### Coaching is an essential element of the adapted OPDG

Patient decision aids, such as the adapted OPDG, facilitate SDM between health care providers and clients [13]. Research has found that the use of patient decision aids needs to be integrated into the process of care [46,47], and increased participation in care can be attained when coaching accompanies the use of patient decision aids [16]. Additionally, health care providers have been encouraged to discuss evidence-based information and to support clients’ chosen level of participation [48].

In our study, Aboriginal women wanted to participate in SDM and identified coaching as an essential element in their decision making process. Coaching used in the individual interviews enabled participants to more fully integrate the adapted OPDG into their decision making process: Specifically, they used the OPDG as a talking guide, used the dialogue with the decision coach as a means to bridge health literacy issues, and found the oral interaction with the decision coach to resonate with their own cultural approach to problem solving. Participants reported that the adapted OPDG with accompanying decision coaching support permitted them to choose their level of involvement in the decision making process and supported a more fulsome engagement in decision making. Of equal importance, our study also found that participants must feel empowered to indicate the *ways* in which they want to be involved. We found that participants expressed a need for an approach that was reflective of their own unique cultural approach to decision making and reflective of who they are as Aboriginal people (that is, First Nations, Métis or Inuit). Such an approach places emphasis on dialogue, community-based decision support and consultation, and the need for a trusted source of information/support. Participants emphasized the importance of the coaching role as a central feature of an effective decision making process, which reflects the cultural importance of relationality, with mutual learning and building of knowledge together. This need for expansion of the coaching role to support women in ways that address the broader context in which they are making health decisions, including empowerment, support, and access to resources has been identified elsewhere [49].

For our study, participants shaped the decision tool and accompanying processes to better support them in their efforts to seek, understand, and use health information to meet their care needs. The approach used in this study was also designed to be culturally resonant by engaging the coach in a collaborative process, to be a ‘trusted’ rather than ‘neutral’ source of support, and to act as an agent with a stake in the process of decision making. There is currently little literature about the health literacy skills of health care providers [18]; however, health care providers have the potential to create positive change at a systems level through the critical exploration of assumptions underlying care systems in collaboration with their Aboriginal clients [2]. In our study, Battiste’s [21] postcolonial theoretical lens showed the ways in which health literacy is influenced by participant adaptations to the OPDG with the role of coaching and the resulting impact on support and/or awareness of individual skills, care systems and broader social, historical, and political contexts. As a tool supporting SDM, the adapted OPDG features the decision coach (a role which may be assumed by trained health care providers who are attuned to building a culturally secure environment) as an essential feature of its use, and as an interactive tool may lead to changes in critical health literacy of health care providers. If so, the result would be additional opportunities to address the unfair processes and issues in existence within care delivery systems, which for Aboriginal people are the results of colonization, and align health care providers with Aboriginal people as equitable partners in reorganizing healthcare.

### Expanding understandings of health literacy

Our findings suggest that standard understandings of health literacy – as a set of skills possessed by an individual [17] – may be influenced by relational factors in the care environment, and specifically, by the relationship with the decision coach. Our work has demonstrated the limitations of applying a normative approach to decision making in which decision makers are encouraged to arrive at health care decisions after a period of self-reflection and an introspective weighing of the personal preferences associated with various options. Instead, our study has demonstrated that participants often prefer to engage in a process of dialogue during which they have the opportunity to articulate the factors underlying their decision making. This dialogue, facilitated by a decision coach, can lead to more collaborative and meaningful discussions and better support decision making solutions that are founded on greater health literacy skills. While our findings show promise for the potential use of SDM tools and approaches for use by and with Aboriginal women, they also suggest that health literacy models may require further examination and expansion. For example, there are other models that have also been developed to understand Aboriginal literacy, such as that of the Rainbow approach [50]; however, the emphasis on health literacy, as opposed to literacy in general, was found to be of particular relevance in our study. Participants were found to be concerned with more than just having information communicated. They placed an emphasis on empowerment of the individual within a health care setting that systematically denied their equitable access to care due to underlying colonial forces. The adaptations to the OPDG and the decision coach may potentially disrupt colonial forces that are evident in health care systems and more accurately reflect the features of health literacy identified as relevant by users, in our case Aboriginal women.

### Limitations and strengths

There are a mix of limitations and strengths to be considered. Findings from the focus groups and usability interviews were from a small group of Aboriginal women; however, participants did self-identify as First Nations, Métis and Inuit, and thus represented a very diverse group. Therefore, these findings have some transferability to other groups. To strengthen the study, the ethical framework, tools and approaches used in the study were developed and approved by and with members of the advisory group that included representatives of the Aboriginal research partner community.

The approach to the adaptation of the OPDG was tailored to meet the needs of those participating in and using the information for the study. The study design incorporated the socio-cultural context of the OPDG users into the process of adaptation and usability testing, an important feature in cross-cultural questionnaires [51]. An additional strength of this study included maintaining the principles underlying the OPDG and therefore the fidelity of the culturally adapted tool [23]. Finally, the second reviewer and members of the advisory group are familiar with the women of Minwaashin Lodge, and were able to verify the study process and findings as relevant to women of Minwaashin Lodge.

## Conclusion

This study describes adaptation and usability testing of the OPDG to support decision making by and with Aboriginal women, and conducted from within a research collaboration inclusive of a particular population of Aboriginal people. Following a process of OPDG adaption using focus groups and then usability interviews, seven themes were developed, reflecting the OPDG adaptations and affirmed the relevance of the adapted OPDG with coaching for this population of Aboriginal women. The major conclusions of our study were that: Adaptations by Aboriginal women to the OPDG resulted in a more accessible version of the original OPDG that was defined by participants as better able to meet their decision making needs; decision coaching was identified as being important to enhance interaction in the use of the adapted OPDG and resulted in the use of the adapted OPDG as a talking guide, and; further research of the adapted OPDG with coaching is required.

The postcolonial theoretical lens was used to show how the adapted OPDG with coaching aligns with the experiences of Aboriginal women as they negotiate complex government or private care institutions. In creating a user-meaningful approach to adaptation of the OPDG and the resulting SDM strategy (adapted OPDG with decision coaching) prominently features user-values as an integral feature. The adapted OPDG with coaching was designed to further support participants’ strengths in the area of health literacy by emphasizing the importance of mutual learning and building of knowledge together with care partners. Our findings show promise for the potential use of SDM tools and approaches for use by and with Aboriginal women; they also suggest that health literacy models may require further examination and expansion to more accurately reflect the features of health literacy identified as relevant by users during the adaptation process, and that are evoked and/or influenced by the decision coach relationship.

In summary, our study has demonstrated a process of adaptation and usability testing of a lower health literacy SDM tool (the adapted OPDG) with decision coaching as an integral feature of its use for fostering engagement in the decision making process. Further collaboration with Aboriginal community partners is needed in research to explore and identify the feasibility and efficacy of using the adapted OPDG with decision coaching as part of effective SDM strategies within Aboriginal populations.
